# Cretaceous/Paleogene Floral Turnover in Patagonia: Drop in Diversity, Low Extinction, and a *Classopollis* Spike

**DOI:** 10.1371/journal.pone.0052455

**Published:** 2012-12-17

**Authors:** Viviana D. Barreda, Nestor R. Cúneo, Peter Wilf, Ellen D. Currano, Roberto A. Scasso, Henk Brinkhuis

**Affiliations:** 1 División Paleobotánica, Museo Argentino de Ciencias Naturales “Bernardino Rivadavia” – CONICET, Buenos Aires, Argentina; 2 Museo Paleontológico Egidio Feruglio – CONICET, Trelew, Chubut, Argentina; 3 Department of Geosciences, Pennsylvania State University, University Park, Pennsylvania, United States of America; 4 Department of Geology and Environmental Earth Science, Miami University, Oxford, Ohio, United States of America; 5 Departamento de Geología, Facultad de Ciencias Exactas y Naturales, Universidad de Buenos Aires, Buenos Aires, Argentina; 6 Institute of Environmental Biology, Faculty of Science, Utrecht University, Utrecht, The Netherlands; The Pennsylvania State University, United States of America

## Abstract

Nearly all data regarding land-plant turnover across the Cretaceous/Paleogene boundary come from western North America, relatively close to the Chicxulub, Mexico impact site. Here, we present a palynological analysis of a section in Patagonia that shows a marked fall in diversity and abundance of nearly all plant groups across the K/Pg interval. Minimum diversity occurs during the earliest Danian, but only a few palynomorphs show true extinctions. The low extinction rate is similar to previous observations from New Zealand. The differing responses between the Southern and Northern hemispheres could be related to the attenuation of damage with increased distance from the impact site, to hemispheric differences in extinction severity, or to both effects. Legacy effects of the terminal Cretaceous event also provide a plausible, partial explanation for the fact that Paleocene and Eocene macrofloras from Patagonia are among the most diverse known globally. Also of great interest, earliest Danian assemblages are dominated by the gymnosperm palynomorphs *Classopollis* of the extinct Mesozoic conifer family Cheirolepidiaceae. The expansion of *Classopollis* after the boundary in Patagonia is another example of typically Mesozoic plant lineages surviving into the Cenozoic in southern Gondwanan areas, and this greatly supports previous hypotheses of high latitude southern regions as biodiversity refugia during the end-Cretaceous global crisis.

## Introduction

The end-Cretaceous mass extinction, resulting from an asteroid impact in the Yucatán at 65.5 Ma, was the most recent great catastrophe for life on Earth [1]–[3]. Land plants may have been less affected than animals, but the only region with abundant, high-resolution stratigraphic data remains the Western Interior USA, relatively close to the Chicxulub crater [4]. There, both macrofloral and microfloral data record significant extinction and a recovery delayed for several million years [4]–[9]. In many sections, diverse latest Cretaceous floras are replaced by Paleocene floras of very low diversity. Many palynofloras from the basalmost Paleocene are dominated by a few species of ferns in a “fern spike” [5], usually interpreted as rapid colonization by pioneer species in a landscape denuded of seed plants.

In the Southern Hemisphere, many marine K/Pg-boundary sections have been recognized, and it is noteworthy that photosynthetic nannoplankton show significantly lower extinction rates and faster recovery in the southern than in the northern hemisphere [10]. To date, there are too few paleobotanical analyses to test whether this pattern is also true of terrestrial photosynthetic organisms, but the data that do exist are consistent with this idea. Sections from Seymour Island (Antarctica) and Australia show little change in the palynological record across the boundary, and no evidence of an abrupt event, although this could be due to relatively coarse sampling or the presence of hiatuses in these records [11], [12]. In contrast, studies from New Zealand, where the K/Pg interval is better defined, show a strong but brief disruption of the vegetation at the boundary, including an initial spike of fungal spores [13], a marked increase in fern spores, a reduction in the relative abundance of gymnosperms, and a temporary loss of angiosperm pollen [14]–[16]. The turnover patterns from New Zealand are completely different from North America, and more like the Southern nannoplankton record, in that no significant species extinctions occurred at, or near, the boundary, and the temporary loss of taxa was followed by an almost complete recovery of the pre-existing Cretaceous elements. In southern South America, early-middle Paleocene macrofloras (ca. 62 Ma) are much more diverse than North American counterparts, suggesting a relatively buffered extinction or faster recovery [17], but these floras date from ca. 4 Ma after the event, and neither macrofloras nor accurately dated palynofloras have previously been compared to latest Cretaceous assemblages in this region.

Here, we explicitly analyze plant turnover in southern South America through palynological examination of a single stratigraphic section in Patagonia from the K/Pg interval. We evaluate the magnitude of vegetation disturbance and examine the role of unexpected colonizer lineages. Although the K/Pg boundary layer itself is not preserved, the patterns nevertheless provide novel insights into geographic variation regarding the end-Cretaceous extinction and recovery.

## Materials and Methods

### Ethics statement

All necessary permits were obtained for the described field studies. Permits were issued by the Secretaría de Cultura de la provincia del Chubut, Argentina.

**Figure 1:**
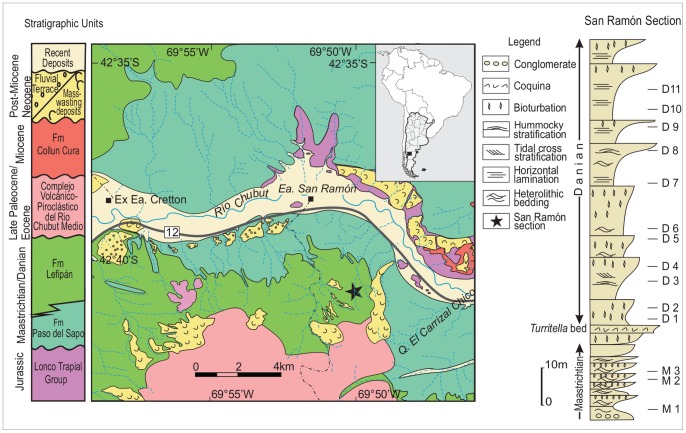
Stratigraphic and geographic location with details of the San Ramón section of the Lefipán Formation (right). Modified from Scasso et al. [16].

The stratigraphic package studied here is the San Ramón section of the Lefipán Formation, which crops out in northwestern Patagonia, Argentina (Figure 1; [18]). The section is up to 400 m thick, of which approximately 260 m are uppermost Cretaceous (Maastrichtian), and the remainder are lowermost Paleocene (Danian). The underlying unit is the Campanian-early Maastrichtian Paso del Sapo Formation, and the overlying unit is the late Paleocene Barda Colorada Ignimbrite. The Lefipán Formation consists of marine to marginal marine, fossiliferous sandstones and mudstones with some intercalated coquinas and conglomerates. Lithofacies analysis indicates the existence of coarse-grained, tide-dominated deltas with sediments input from braided rivers, formed at the western margin of a large marine embayment during the K/Pg boundary interval [18]. In the middle part of the sequence, a coquina bed rich in the gastropods *Turritella* and/or *Pseudamaura* marks the earliest Paleocene deposits in the area and represents a transgressive accumulation produced by wave-induced oscillatory flows, in a high-energy, shallow marine environment (Figure 1). The uppermost Maastrichtian is represented by tidal channel and tidal flat deposits with a low diversity *Corbicula* assemblage indicative of low salinity, high-stress environments unfavorable to most marine species that characterize the stable, normal marine, Maastrichtian infaunal guilds in most of the succession. During the early Danian coarsening-upward cycles reflect accumulation in tidal bars, mainly in a tide to wave-influenced distal delta-front environment with high sedimentation rates. These deposits bear molluscan faunal associations indicative of recurrent changes from normal marine to low-salinity marine environments with low-oxygen, fine-grained, soupy substrates [18].

The identification of the K/Pg interval in the studied section (San Ramón) is based on three criteria. First, there is a major change in invertebrate faunal composition within a few meters of section wherein two successive, diagnostically Maastrichtian faunas are replaced by a succession of two Danian assemblages [18]–[20]. Second, latest Maastrichtian and earliest Danian dinoflagellate markers are present in the corresponding strata, including *Cyclapophysis monmouthensis* and *Damassadinium californicum*, respectively. Third, age-diagnostic continental palynomorphs are present in the corresponding beds, including the late Maastrichtian markers *Grapnelispora evansii, Quadraplanus brossus*, and *Tubulifloridites lilliei*, and the Danian marker *Nothofagidites dorotensis*. The boundary layer itself is apparently not preserved, but the biostratigraphic data indicate only a minimal temporal hiatus, and we consider the most likely bracket for the boundary interval to be approximately 4 m of strata within an 8.7 m thick, highly bioturbated, fine to medium-grained sandstone (Figure 1).

Fourteen outcrop samples were analyzed spanning 90 meters of the middle-upper Lefipán Formation. Samples of variable palynological content and preservation were obtained as close as 6.3 m to the suggested K/Pg boundary interval, on either side (Figure 1). Samples were prepared using standard palynological techniques, and most contained both marine (dinoflagellate cysts) and continental (spores, pollen grains, fungal remains, fresh water algae) palynomorphs. Pollen and spores were identified to species level whenever possible. A minimum of 300 continental specimens were counted in each sample, except in samples M1, M3 and D13, for a total of 5887 counted specimens. Slides are housed in the palynological collection of the Museo Paleontológico Egidio Feruglio, Trelew, Chubut, Argentina, under the prefix MPEF-Palin, numbers 100 to 113. Coordinates of the illustrated specimens refer to the England finder. Pollen terminology follows Punt et al. [21]. Groups of samples were determined by CONISS [22] stratigraphically constrained cluster analysis. The TGVIEW program was used for cluster analysis and plotting diagrams [22]. All other quantitative analyses were done using the R program, version 2.2.0 (http://www.r-project.org). A nonmetric multi-dimensional scaling analysis (NMDS) was used to ordinate the pollen and spore data. The data were arcsine square root transformed to improve normality [23], a dissimilarity matrix was computed using the Bray-Curtis distance metric [24], and R's “metaMDS” function performed the NMDS ordination. Diversity within a sample was estimated using the Shannon-Wiener Index and analytical rarefaction. Pielou's J metric was used to compare evenness among samples.

## Results

One hundred and thirty spore and pollen species were recovered (Table S1), of which 40 were previously reported from the lower part of the Lefipán Formation [25], [26].

**Figure 2:**
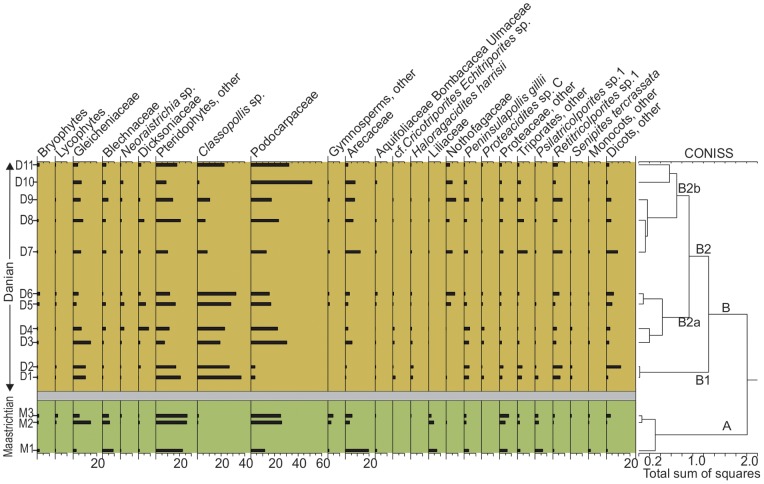
Spore and pollen diagram shown as percent abundances per sample from the Lefipán Formation. The CONISS cluster dendrogram at right determines distinct, stratigraphically ordered palynomorph groups. Note large Danian increase in *Classopollis* abundance.

Cluster analysis (Q mode) revealed two stratigraphically well-ordered major groups of samples (Figure 2). Group A comprises the Maastrichtian samples (M1–M3) and Group B all early Danian samples, with two subgroups: B1 (samples D1 and D2) and B2 (samples D3–D11). Group A (Maastrichtian) is dominated by fern spores (mean 41%), followed by angiosperms (mean 34%) and gymnosperms (mean 24%; Table S2). Spores of bryophytes and lycophytes are uncommon (1.5%) but present in most Cretaceous samples (Table S1; Figure 2). Ferns are represented mainly by Gleicheniaceae, *Cyathidites* spp. (uncertain affinity), and Blechnaceae. There are also occurrences of aquatic ferns (Azollaceae). The most abundant angiosperm taxa are Arecaceae (including *Nypa* palms), Proteaceae, Liliaceae, and *Psilatricolporites* sp. (uncertain affinity). There are also low-frequency occurrences of Aquifoliaceae, Malvaceae (Bombacoideae), Chloranthaceae, and some monocots (Sparganiaceae). Gymnosperms are largely represented by Podocarpaceae (*Podocarpus* type), and other gymnosperms (Araucariaceae, extinct coniferophyte, and Cycadales–Bennettitales–Ginkgoales) occur at lower abundances.

Group B1 (basalmost Danian) is dominated by the gymnosperm *Classopollis* ( = *Corollina*) of the extinct conifer family Cheirolepidiaceae, which reaches up to 37% of the total pollen content, sharply up from less than 1% in latest Maastrichtian samples (Table S1, Figure 2). Other gymnosperms are scarce (3.5%) and exclusively represented by Podocarpaceae. Ferns (32%) decrease in both relative abundance and diversity: terrestrial Gleicheniaceae and *Cyathidites* spp. are the most abundant, but less so than during the Maastrichtian. Angiosperms (27%) are mainly represented by *Penninsulapollis gilli* (Proteaceae related to *Beauprea*), *Retritricolporites* sp. 1 (uncertain affinity), *Haloragacidites harrisii* (Casuarinaceae), and other triporate species (*Cicotriporites* sp., *Echitriporites* sp., *Triatriopollenites* spp.). Interestingly, some taxa well documented in Maastrichtian samples, particularly those of Liliaceae, Arecaceae, and some Proteaceae, suffered a general reduction in abundance in the basalmost Danian samples (Table S1).

Group B2 is divided into two subgroups B2a and B2b (Figure 2). These are both dominated by gymnosperms (mean 42.5%), including more than five species of Podocarpaceae and *Classopollis*, the latter showing a broad decline in abundance towards the levels containing subgroup B2 b (Table S1, S2; Figure 2). Angiosperms occupy the second rank in abundance (mean 30%), and prevalent elements include Nothofagaceae and Ericaceae. Some Arecaceae (including *Nypa*), several Proteaceae, Liliaceae, Aquifoliaceae, and Malvaceae (Bombacoideae) are recorded again, or have higher frequencies than before, in levels containing the B2 cluster. This trend is even more pronounced in subgroup B2b. There, ferns decrease to a mean value of 27% of the assemblages with an increased participation of ?Osmundaceae (*Neoraistrichia* sp.), and Dicksoniaceae (*Trilites* spp.), but the relative abundance of ferns remains lower than in group A; bryophytes are uncommon (1%) and lycophytes extremely scarce.

**Figure 3:**
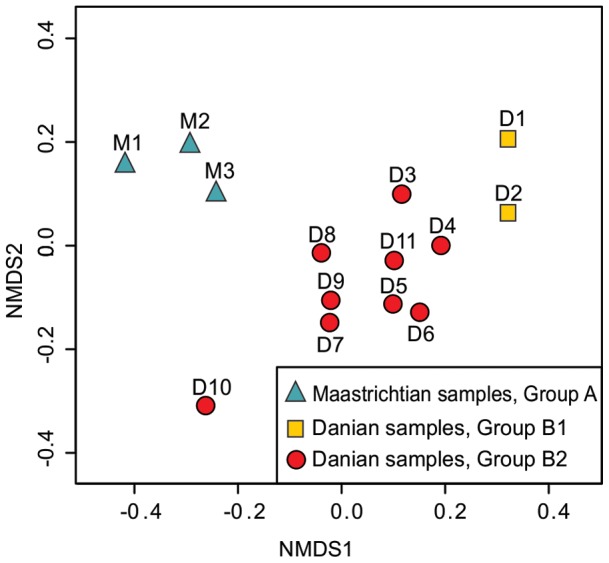
Results of non-metric multidimensional scaling (NMDS) ordination of spore-pollen data from the Lefipán Formation. Sample numbers and cluster groups as in Figure 2. Stress = 9.99.

The NMDS analysis supports the clustering of samples defined above (Figure 3), again clearly separating the earliest Danian samples (D1–D2, Group B1) from those of the Maastrichtian (M1–M3, Group A). Samples D3 to D11 (Group B2) occupy an intermediate position between the Maastrichtian samples (M1–M3) and those of the earliest Danian (D1–D2).

**Figure 4:**
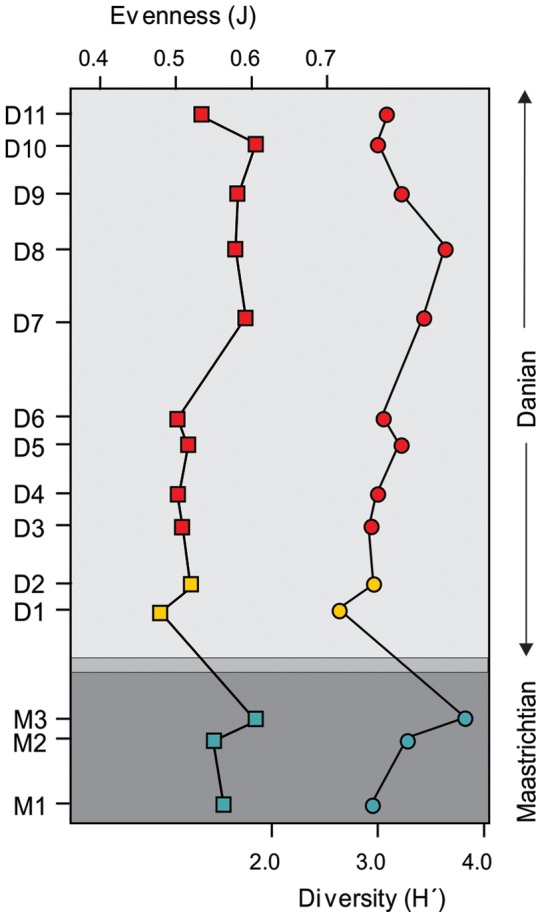
Spore-pollen evenness (Pielou's *J*, left) and diversity (Shannon-Wiener Index, H', right), from the Lefipán Formation. (Figure 1).

**Figure 5:**
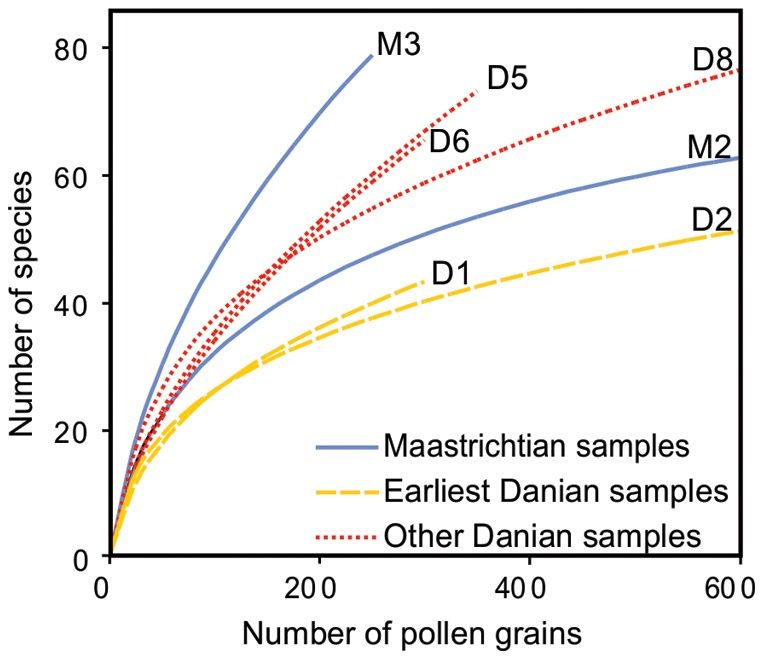
Rarefaction results from spore-pollen counts of selected samples from the Lefipán Formation (**Figure** **1**).

Measures of diversity (Shannon-Wiener index, rarefaction) and evenness (Pieleu's J) show a marked decrease from the latest Cretaceous to the Danian, and a minimum value in the lowest Danian sample (Figure 4, 5). The highest richness occurs in Sample M3, the uppermost Maastrichtian sample that yielded abundant palynomorphs, about 12.6 m below the Danian marker bed (Figure 4, 5). The rarefied richness at 250 specimens is 78 species in Sample M3 (Figure 5), whereas the lowest values of rarefied species richness occur in the first 14 m of the Danian (Samples D1 to D2) and range from 37 to 47 species. The rarefaction curves clearly indicate that a sustained recovery in diversity occurs at about 30 m above the boundary (sample D5) where several characteristic “Cretaceous species” re-appeared (ie. *Echinosporis* sp., *Reticuloidosporites tenellis, Trisaccites microsaccatum, Araucariacites australis, Liliacidites variegatus*, *Nothofagidites saraensis*) and other species are recorded for the first time (ie. *Herkosporites elliottii, Trilites parvallatus, Nothofagidites fuegiensis, Propylipollis* cf. *tenuiexinus, Psilatricolporites salamanquensis*, *Striatricolporites gamerroi, Tricolpites* sp. 1). Evenness sharply decreases across the K/Pg interval and increases again at Sample D7 (Figure 4).

## Discussion and Conclusions

Our results provide the first outline, on the basis of spores and pollen grains, of vegetational history across the K/Pg interval in central Patagonia. The analyses indicate a major floristic shift and decline in richness at the beginning of the Paleocene, but not a great extinction event.

**Figure 6:**
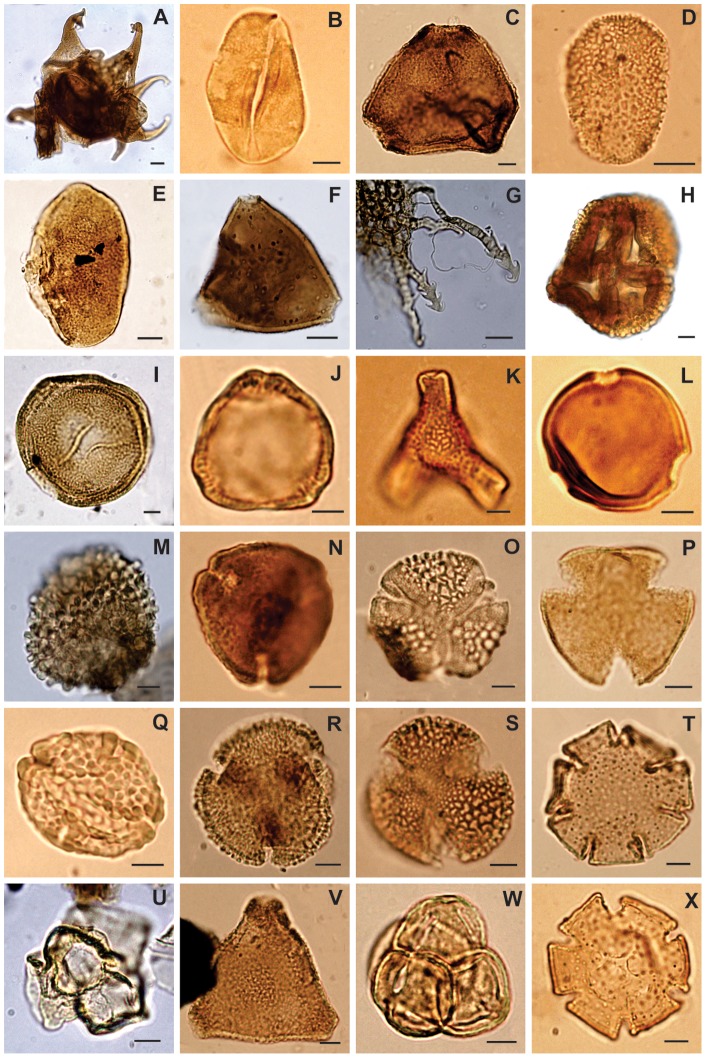
Selected characteristic species from the Maastrichtian (A–H) and Danian (I–X) samples. Scale bar equals 5 µm, except in figures A, E, F and G where scale bar is equal 10 µm. Authorities are given in Table S1. A, *Grapnelispora evansii* (MPEF–Palin 102c: V32–4); B, *Arecipites minutiscabratus* (MPEF–Palin 101b: Q31–1); C, *Lewalanipollis senectus* (MPEF–Palin 101a: S47); D, *Liliacidites regularis* (MPEF–Palin 101b: N24–1); E, *Longapertites* aff. *vaneendenburgi* (MPEF–Palin 102a: P29); F, *Propylipollis ambiguus* (MPEF–Palin 102a: K33–1); G, *Azollopsis tormentosa* (MPEF–Palin 102a: Q33–4); H, *Quadraplanus brossus* (MPEF–Palin 102d: N37–1); I, *Classopollis* sp. (MPEF–Palin 103a: D43–4); J, *Haloragacidites harrisii* (MPEF–Palin 107b: F49); K, *Proteacidites* sp. C (MPEF–Palin 107b: P39–1); L, cf. *Cicotriporites* sp. (MPEF–Palin 103b: D33–1); M, *Neoraistrichia* sp. A (MPEF–Palin 103a: N39–3); N, *Senipites tercrassata* (MPEF–Palin 103b: M29); O, *Retritricolporites* sp. 1 (MPEF–Palin 108b: Y26–3); P, *Peninsulapollis gillii* (MPEF–Palin 109b: N28–4); Q, *Ulmoideipites patagonicus* (MPEF–Palin 108 b: M49–3); R, *Bombacacidites* cf. *isoreticulatus* (MPEF–Palin 108 b: L51–2); S, *Rousea microreticulata* (MPEF–Palin 104b: Z35/Z36); T, *Nothofagidites dorotensis* (MPEF–Palin 107a: V37); U, *Rosannia manika* (MPEF–Palin 106 b: W45); V, *Propylipollis reticuloscabratus* (MPEF–Palin 110d: M36–1); W, *Ericipites microtectatum* (MPEF–Palin 110d: F38–3); X, *Nothofagidites fuegiensis* (MPEF–Palin 109 b: F23–4).

**Figure 7:**
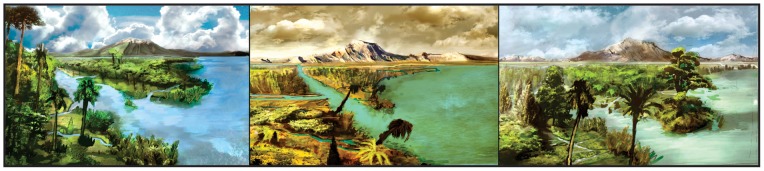
Ancient landscapes across the Cretaceous/Paleogene time interval in central Patagonia. Main floristic types from the: Maastrichtian (left): Ferns, palms, conifers; earliest Danian (middle): Cheirolepidiaceae, shrubs and other low–diversity flora; and Danian (right): Podocarps, Cheirolepidiaceae, palms.

As understood here, the presence of a fern-angiosperm dominated community during the Maastrichtian, with gymnosperms (podocarps) as common trees, diverse Proteaceae, aquatic ferns, and abundant palms suggests warm and humid adapted vegetation. Clearly, palms played a significant role in late Maastrichtian communities (Figure 6A–H, 7). The presence of *Spinizonocolpites*, related to the tropical mangrove palm *Nypa*, indicates specialized shore-line mangrove assemblages. Proteaceae related to *Beauprea* and *Telopea*, together with Aquifoliaceae, may have formed a lower stratum of forests or woodlands. The abundant monocots, primarily Liliaceae and some Sparganiaceae, Chloranthaceae and the diverse ferns may have grown in the understory, associated with ponds, small streams or rivers just landward of the shoreline.

Earliest Danian vegetation (Group B1) had low diversity (Figure 4, 5, 7) and was quite different in species composition and abundance from both the latest Maastrichtian (Group A) and the subsequent Danian (Group B2) assemblages (Figure 2, 3). The marked reduction in diversity affected all groups of plants, particularly ferns and monocots, most Proteaceae species except *Beauprea*-like form, and nearly all gymnosperms. The presence of marginal, shallow marine, somewhat stressed paleoenvironmental conditions on both sides of the K/Pg boundary indicates that changing depositional factors were unlikely to have had significant importance in driving the observed compositional changes in the palynological assemblages. Earliest Danian assemblages were characterized by the striking abundance of *Classopollis*, a pollen type linked to Casuarinaceae *(Haloragacidites harrisii*), the consistent presence of *Beauprea* (*Peninsulapollis gillii*), ferns of Gleicheniaceae (Figure 6I–P) and some taxa of unknown affinity (*Retritricolporites* sp. 1, *Cicotriporites* sp., *Echitriporites* sp., *Triatriopollenites* spp.). Moreover, the low values of evenness recorded in Sample D1 are consistent with a highly disturbed flora. Most early Danian taxa are not well understood in terms of their ecological requirements, but the common feature of many members of this group of plants is their notable adaptability to changing environments. The association of *Classopollis* with stressed (disturbed) environments is well known [27].

Later Danian vegetation (Group B2) was dominated by gymnosperms, including diverse Podocarpaceae related to *Podocarpus, Microcachrys*, *Dacrydium, Lagarostrobos* and *Dacrycarpus. Classopollis* remained abundant, but it shows a general reduction towards younger samples. Palms and tree ferns of Dicksoniaceae were also important components of the Danian vegetation. Other elements included *Nothofagus*, diverse eudicots of uncertain affinity, and new species of Proteaceae and Ericaceae, indicating that several typical components of extant austral forests were in place by the Danian (Figure 6Q–X, 7). Several Cretaceous taxa return again in this part of the sequence including Liliaceae and temperate to warmth-loving families: Aquifoliaceae, Malvaceae (Bombacoideae) and Arecaceae (*Nypa* type). The presence of Lactoridaceae, a monotypic family today restricted to subtropical forests of the Juan Fernández Archipelago, offshore from Chile in the South Pacific Ocean, is particularly striking. These assemblages are fairly diverse, although no Danian sample reaches the diversity recorded in the latest Maastrichtian (Figure 5, 6).

In broad terms, this analysis shows a reduction in diversity and relative abundance in almost all plant groups from the latest Maastrichtian to the Danian, although only a few true extinctions occurred. Many of the species that disappear at the boundary return higher in the sequence, indicating their survival in refugial areas. The overall extinction rate is difficult to estimate without more exhaustive studies, but it probably does not exceed 10% of the species. This pattern of recovery is comparable to that observed in New Zealand, where an abrupt disturbance of the vegetation across the K/Pg boundary occurred, but with a low overall extinction rate [14]–[16].

The different floral devastation patterns recorded in North America and the southern Hemisphere, now including Patagonia, may be related to the attenuation of damage with increased distance from the impact site, following the global pattern of decreasing sediment disturbance with increasing distance from Chicxulub [3]. This hypothesis is supported by paleobotanical data from the Paleocene of Colombia, which was near the impact site and appears to show a depressed ecosystem, and from France, which shows the opposite pattern [28], [29]. Alternatively, northern vs. southern hemispheric differences may have been the dominant effect, as shown in the global nannoplankton data [10]. Because Patagonia is very distant from Chicxulub and is in the Southern Hemisphere, our results are compatible with both hypotheses, alone or in combination. Our data also suggest that the elevated richness observed in Paleocene and Eocene macrofloras from Patagonia [17], [30] may, in part, be a legacy effect of a muted K/Pg extinction event.

The peak of *Classopollis* just above the K/Pg boundary is hypothetically a temporal correlate of the classic, short-lived fern spike seen elsewhere. However, there is not sufficient temporal resolution in the San Ramon section to determine whether a fern spike was absent versus non-sampled, and thus whether the *Classopollis* increase followed, or locally replaced, the widely distributed fern spike. Cheirolepidiaceae is a common component of Mesozoic floras, having last records in most regions in the Cenomanian and Turonian [27], [31], [32]. In southern South America, however, *Classopollis* showed a somewhat different behavior, reappearing in the record in the latest Maastrichtian and playing a significant role in Paleocene plant communities [33]–[36]. Even though it has not yet been found in situ in a Paleocene pollen cone, the *Classopollis* pollen type (Figure 6I) is so distinctive that it is very unlikely to belong to another group of plants besides Cheirolepidiaceae. Reworking has been sometimes invoked to explain comparable occurrences [31]. However, the Paleocene *Classopollis* are not corroded or otherwise degraded, showing no significant difference in color in comparison from the rest of the assemblages that would support reworking. Moreover these palynomorphs have been recorded in several Paleocene Argentinean basins occupying different paleoenvironments and at high relative abundances [33]–[36]. Thus, we find no evidence that Paleocene *Classopollis* was recycled from older strata, and instead we conclude that this genus persisted longer in southern South America than elsewhere, until its final Paleocene appearance. The terminal Cretaceous event caused significant changes in terrestrial ecosystems that appear to have favored the regeneration and spread of *Classopollis* in southernmost South America. Newly disturbed habitats with few competitors may have created suitable ecospaces for the re-establishment of this conifer, adapted to stressed environments [27].

It is also noteworthy that other dominant “Mesozoic” plant lineages considered to have disappeared globally at the end of the Cretaceous survived during the Paleogene in southern Gondwana. Particularly striking is the record of leaves resembling corystosperms in the Paleogene of Tasmania [37], along with the presence of extinct clades of Ginkgoales in Tasmania [38] and in Patagonia [39], [40]. Other records of this type include bennettitaleans from the Oligocene of Australia, following an extraordinarily long stratigraphic hiatus of more than 50 Ma [41]. Moreover, *Araucaria* is another interesting case; it shows a nearly worldwide distribution through the Mesozoic, but it disappeared from North America and Europe in the latest Cretaceous, continuing in the Southern Hemisphere only during the Cenozoic, where it has a relict distribution today [42], [43]. It is also remarkable that many groups of Cretaceous Patagonian vertebrates survived into the Paleogene. This is the case for several marsupial taxa that successfully crossed the K/Pg boundary in South America, while this group was severely reduced in North America [44]. The same is true for a dryolestoid mammal recorded in Patagonia in the Paleocene as a survivor of a Cretaceous South American radiation [45].

The expansion of *Classopollis* in Patagonia after the terminal Cretaceous event, together with a growing list of Mesozoic lineages persisting into the Cenozoic in southern Gondwanan areas, greatly supports the hypothesis of southern high-latitude regions as biodiversity refugia from the global environmental crisis at the end of the Cretaceous [37].

## Supporting Information

Table S1 (DOCX)

Table S2 (DOCX)
