# Supplementary material for: Cretaceous/Paleogene Floral Turnover in Patagonia: Drop in Diversity, Low Extinction, and a Classopollis Spike
Source: PLoS One. 2012 Dec 17;7(12):e52455. doi: 10.1371/journal.pone.0052455 (PMC3524134; doi:10.1371/journal.pone.0052455)
Supplement: Table S1 — (DOCX) [file pone.0052455.s001.docx]

**Table S1**. Species list, botanical affinities and proportional abundances

| Fossil taxon | Nearest living relatives | Proportional abundances | | | | | | | | | | | | | |
| --- | --- | --- | --- | --- | --- | --- | --- | --- | --- | --- | --- | --- | --- | --- | --- |
|  |  | M1 | M2 | M3 | D1 | D2 | D3 | D4 | D5 | D6 | D7 | D8 | D9 | D10 | D11 |
| Bryophyte spores |  |  |  |  |  |  |  |  |  |  |  |  |  |  |  |
| *Rhabdosporites* sp. |  | 0 | 0 | 0 | 0 | 0 | 0 | 0 | 0.281 | 0.321 | 0 | 0.158 | 0 | 0 | 0 |
| *Rouseisporites* sp. | Ricciaceae | 0 | 0 | 0 | 0 | 0 | 0 | 0 | 0 | 0 | 0 | 0 | 0 | 0 | 0.267 |
| *Stereisporites* (*Tripunctisporis*) sp. | Sphagnaceae | 0 | 0.327 | 0.389 | 0.308 | 0.326 | 0 | 0.097 | 0.562 | 0 | 0 | 0 | 0 | 0 | 0 |
| *Stereisporites antiquasporites* (Wilson & Webster) Dettmann 1963 | Sphagnaceae | 1.351 | 0.164 | 0.778 | 0 | 0 | 0.241 | 0 | 0.281 | 0.641 | 0 | 0.317 | 0 | 0 | 0.267 |
| *Stereisporites regium* (Drozhastchich) Drugg 1967 | Sphagnaceae | 0 | 0 | 0 | 0 | 0 | 0 | 0 | 0 | 0.321 | 0 | 0 | 0 | 0 | 0 |
| *Stereisporites* sp. 2 | Sphagnaceae | 0 | 0 | 0 | 0 | 0 | 0.241 | 0 | 0.562 | 0.641 | 0.368 | 0.317 | 0 | 0 | 0.802 |
| Lycophyte spores |  |  |  |  |  |  |  |  |  |  |  |  |  |  |  |
| *Camarozonosporites ohaiensis* (Fradkina) Playford 1971 | Lycopodiaceae | 0 | 0 | 0.389 | 0 | 0 | 0 | 0 | 0 | 0 | 0 | 0 | 0 | 0 | 0 |
| *Camarozonosporites* spp. | Lycopodiaceae | 0 | 0.327 | 0.389 | 0.308 | 0.651 | 0 | 0.389 | 0 | 0 | 0.184 | 0.317 | 0 | 0 | 0 |
| *Ceratosporites equalis* Cookson & Dettmann 1958 | Selaginellaceae | 0 | 0.164 | 0.389 | 0 | 0.326 | 0 | 0.097 | 0.281 | 0.321 | 0 | 0 | 0 | 0 | 0 |
| *Densoisporites velatus* Weyland & Krieger 1953 | Selaginellaceae | 0 | 0.164 | 0.389 | 0 | 0 | 0 | 0 | 0 | 0 | 0 | 0 | 0 | 0 | 0 |
| *Herkosporites elliottii* Stover 1973 | Selaginellaceae | 0 | 0 | 0 | 0 | 0 | 0 | 0 | 0.281 | 0.321 | 0 | 0 | 0 | 0 | 0 |
| *Perotrilites* sp. | Selaginellaceae | 0 | 0 | 0 | 0 | 0 | 0 | 0 | 0.281 | 0 | 0 | 0 | 0 | 0 | 0.267 |
| *Retitriletes austroclavatidites* (Cooks.) Dör.. Kr. Mai & Schulz 1963 | Lycopodiaceae (*Lycopodium*) | 0.450 | 0.164 | 0.389 | 0.308 | 0 | 0 | 0 | 0 | 0 | 0.184 | 0.158 | 0.322 | 0 | 0 |
| Pteridophyte spores |  |  |  |  |  |  |  |  |  |  |  |  |  |  |  |
| *Azollopsis tormentosa* Hall 1968 | Azollaceae | 0 | 0 | 0.389 | 0 | 0 | 0 | 0 | 0 | 0 | 0 | 0 | 0 | 0 | 0 |
| *Baculatisporites comaumensis* (Cookson) Potonié 1953 | Hymenophyllaceae? | 0 | 0 | 1.167 | 0 | 0 | 0 | 0 | 0 | 0 | 0 | 0 | 0 | 0 | 0 |
| *Biretisporites* sp. | unknown fern | 0 | 0 | 0.778 | 0 | 0 | 0 | 0 | 0.281 | 0 | 0 | 0 | 0 | 0 | 0 |
| *Cicatricosisporites* sp. | Schizaeaceae | 0.450 | 0 | 0 | 0 | 0.326 | 0 | 0 | 0 | 0 | 0 | 0.158 | 0 | 0 | 0 |
| *Clavifera triplex* (Bolkhovitina) Bolkhovitina | Gleicheniaceae | 0 | 1.309 | 1.167 | 0.308 | 0.651 | 0.241 | 0.097 | 0 | 0.321 | 1.471 | 1.585 | 0 | 1.036 | 0.267 |
| *Convolutispora* sp. 1 | Unknown fern | 0.450 | 0 | 0.389 | 0.923 | 0.326 | 0 | 0.583 | 0 | 0 | 0 | 1.585 | 0 | 0.518 | 0.535 |
| *Convolutispora* sp. 2 | Unknown fern | 0 | 0.164 | 0 | 0 | 0 | 0 | 0 | 0 | 0 | 0 | 0 | 0 | 0 | 0 |
| *Cyatheacidites annulatus* Cookson 1947 | Lophosoriaceae (*Lophosoria*) | 0.450 | 0.655 | 0.389 | 0.308 | 0.326 | 0.241 | 0.097 | 0 | 0 | 0 | 0.158 | 0 | 0 | 0.802 |
| *Cyathidites australis* Couper 1953 | Unknown fern | 14.865 | 2.619 | 3.891 | 0.308 | 3.257 | 1.446 | 0.097 | 6.180 | 2.244 | 5.882 | 7.448 | 0.965 | 6.218 | 1.872 |
| *Cyathidites minor* Couper 1953 | Unknown fern | 0 | 7.038 | 4.669 | 3.077 | 0.326 | 0.723 | 3.790 | 7.303 | 8.013 | 2.022 | 1.426 | 2.894 | 0 | 3.743 |
| *Cyathidites patagonicus* Archangelsky 1972 | Unknown fern | 0 | 0.818 | 0.778 | 2.462 | 0.651 | 2.169 | 0.097 | 0 | 0 | 1.654 | 3.487 | 0.322 | 0 | 6.417 |
| *Deltoidospora* sp. | Unknown fern | 5.405 | 11.129 | 4.280 | 9.231 | 7.818 | 1.205 | 5.734 | 0.281 | 0.321 | 0 | 3.962 | 10.289 | 0 | 1.337 |
| *Echinosporis* sp. | Unknown fern | 0.450 | 0.327 | 0.778 | 0 | 0 | 0 | 0.194 | 0.562 | 0 | 0 | 0.158 | 0 | 0 | 0 |
| *Foveotriletes* sp. cf. *F.* *parvirectus* (Balme) Dettmann | Lycopodiaceae | 0 | 0.491 | 0.778 | 1.231 | 0.326 | 0.723 | 0.097 | 0.281 | 0.321 | 0 | 1.109 | 0 | 1.036 | 0.802 |
| *Gleicheniidites circinidites* (Cookson) Dettmann 1963 | Gleicheniaceae | 0 | 0 | 0 | 0.308 | 0 | 0 | 0 | 0.281 | 0.321 | 0 | 0.951 | 1.286 | 0 | 0.267 |
| *Gleicheniidites senonicus* Ross 1949 | Gleicheniaceae | 2.703 | 13.257 | 3.891 | 9.231 | 8.795 | 14.217 | 6.900 | 2.247 | 3.846 | 4.963 | 5.705 | 5.145 | 6.218 | 3.476 |
| *Gleicheniidites* sp. | Gleicheniaceae | 0 | 0.327 | 0.389 | 0.615 | 0.977 | 0.241 | 0.097 | 0.281 | 0.000 | 0 | 0 | 0 | 0 | 0 |
| *Grapnelispora evansii* Stover & Partridge 1984 | Salviniaceae? | 0 | 0 | 0.389 | 0 | 0 | 0 | 0 | 0 | 0 | 0 | 0 | 0 | 0 | 0 |
| *Laevigatosporites ovatus* Wilson & Webster 1946 | Blechnaceae | 9.009 | 5.728 | 5.058 | 0.308 | 0.651 | 0.723 | 2.430 | 2.528 | 0.321 | 2.941 | 2.536 | 4.502 | 2.591 | 2.674 |
| *Leiotriletes* sp. | Unknown fern | 0 | 1.146 | 4.280 | 0.923 | 0 | 0 | 0.194 | 0 | 0 | 0.184 | 0 | 0 | 0 | 0.267 |
| *Matonisporites* sp. | Dicksoniaceae | 0 | 0 | 0 | 0 | 0.977 | 0 | 0.194 | 0.281 | 0 | 0 | 0.634 | 0 | 0 | 0 |
| *Microfoveolatosporites* sp. | Unknown fern | 0 | 0.164 | 0.778 | 0 | 0 | 0.24 | 0.10 | 0 | 0 | 0 | 0.158 | 0 | 0 | 0 |
| *Neoraistrichia* sp. of Archangelsky 1973 | Osmundaceae? | 0.450 | 0.164 | 0.778 | 0.308 | 0.326 | 0.964 | 3.207 | 3.090 | 1.282 | 1.654 | 0.951 | 1.286 | 2.073 | 0.267 |
| *Peromonolites* sp. 2 | Blechnaceae? | 0 | 0 | 0.389 | 0 | 0 | 0 | 0.097 | 0 | 0 | 0 | 0 | 0 | 0 | 0.267 |
| *Peromonolites* sp.1 | Blechnaceae? | 0 | 0.327 | 0.389 | 0 | 0 | 0 | 0 | 0 | 0 | 0 | 0 | 0 | 0 | 0.267 |
| *Peromonolites vellosus* Partridge 1973 | Blechnaceae | 0 | 0.164 | 0.389 | 0 | 0 | 0 | 0 | 0.281 | 0.321 | 0 | 0 | 0 | 0 | 0 |
| *Polypodiaceoisporites* sp. | Pteridaceae | 0 | 0 | 0 | 0 | 0 | 0 | 0 | 0 | 0.321 | 0 | 0 | 0 | 0 | 0 |
| *Polypodiidites speciosus*  (Harris) Archangelsky 1972 | Polypodiaceae | 0 | 0 | 0 | 0 | 0.326 | 0.241 | 0 | 0.281 | 0 | 0 | 0 | 0 | 0 | 0 |
| *Reticuloidosporites tenellis*Krutzsch 1959 | Polypodiaceae | 0 | 0.164 | 0 | 0 | 0 | 0 | 0 | 0.281 | 0 | 0 | 0.158 | 0 | 0 | 0 |
| *Rugulatisporites chubutensis* Baldoni 1992 | Unknown fern | 0 | 0 | 0.389 | 0.308 | 0.326 | 0.241 | 0.097 | 0 | 0 | 0 | 0.158 | 0 | 1.036 | 0 |
| *Sestrosporites pseudoalveolatus* (Couper) Dettmann 1963 | Lycopodiaceae | 0 | 0.818 | 0.389 | 0 | 0.326 | 0 | 0 | 0 | 0 | 0 | 0 | 0 | 0 | 0 |
| *Trilites parvallatus* Krutzsch 1959 | Dicksoniaceae? | 0 | 0 | 0 | 0 | 0 | 0 | 0 | 0.843 | 0 | 0 | 0.475 | 0.965 | 0 | 0 |
| *Trilites* sp. 1 | Dicksoniaceae? | 0.450 | 0.818 | 0.389 | 0 | 0 | 0 | 0 | 0 | 0 | 0 | 0 | 0 | 0 | 0 |
| *Trilites* sp. 2 | Dicksoniaceae? | 0 | 0 | 0 | 0 | 0.326 | 0.964 | 4.762 | 0 | 0.321 | 0 | 0 | 0 | 0 | 0 |
| *Trilites tuberculiformis* Cookson 1947 | Dicksoniaceae | 0 | 0 | 0 | 0 | 0 | 0 | 2.624 | 3.933 | 0.962 | 1.654 | 1.743 | 0 | 0 | 1.337 |
| *Trilites volkheimerii* Filatoff 1975 | unknown fern | 0.450 | 0.164 | 0.389 | 0.308 | 1.629 | 0.723 | 1.361 | 1.404 | 0.641 | 0.184 | 2.536 | 0.643 | 0 | 1.070 |
| *Verrucosisporites* sp. 1 | Unknown fern | 0 | 0 | 0.389 | 1.538 | 1.629 | 0 | 0 | 0 | 0 | 0 | 0 | 0 | 0 | 0.802 |
| *Verrucosisporites* sp. 2 | Unknown fern | 0 | 0 | 0 | 0 | 0 | 0 | 0 | 0 | 0 | 0 | 0.158 | 0 | 0 | 0.267 |
| Gymnosperm pollen |  |  |  |  |  |  |  |  |  |  |  |  |  |  |  |
| *Araucariacites australis* Cookson 1947 | Araucariaceae (*Araucaria*) | 0.901 | 0.327 | 0.389 | 0 | 0 | 0 | 0 | 0.562 | 0.641 | 0 | 0.951 | 0.322 | 0 | 0 |
| *Classopollis* sp. | Extinct (Cheirolepideaceae) | 0 | 0.327 | 0.778 | 36.923 | 26.710 | 19.277 | 22.935 | 27.809 | 32.051 | 8.456 | 6.656 | 10.289 | 1.554 | 22.193 |
| *Cycadopites* sp. | Cycadaceae | 0 | 0.327 | 0.389 | 0 | 0 | 0 | 0 | 0 | 0 | 0 | 0 | 0 | 0.518 | 0 |
| *Dacrycarpites australiensis* Cookson & Pike 1953 | Podocarpaceae (*Dacrycarpus*) | 0 | 0 | 0 | 0 | 0 | 0 | 0.097 | 0.281 | 0.321 | 0 | 0 | 0 | 0 | 0 |
| *Gamerroites psilasaccus* (Archangelsky & Romero) Archangelsky 1988 | Podocarpaceae | 0 | 0.327 | 1.167 | 0 | 0 | 0.241 | 0.389 | 0.281 | 0.962 | 0 | 0.475 | 0.322 | 3.627 | 1.070 |
| *Lygistepollenites florinii* (Cookson & Pike) Stover & Evans 1973 | Podocarpaceae (*Dacrydium*) | 0 | 0.327 | 0.389 | 0 | 0 | 0.241 | 0.292 | 1.685 | 0.641 | 0 | 2.377 | 0 | 2.073 | 1.070 |
| *Microcachryidites antarcticus* Cookson 1947 | Podocarpaceae (*Microcachrys*) | 0.901 | 3.437 | 0.778 | 0.308 | 0 | 0.241 | 0.292 | 0.281 | 0.641 | 0.368 | 0.158 | 0.643 | 2.073 | 0.267 |
| *Phyllocladidites mawsonii* Cookson 1947 | Podocarpaceae (*Lagarostrobos*) | 0.901 | 3.273 | 2.724 | 0.615 | 1.303 | 6.747 | 5.734 | 3.371 | 0.641 | 2.390 | 6.339 | 2.251 | 6.218 | 5.348 |
| *Podocarpidites elegans* Romero 1977 | Podocarpaceae (*Podocarpus*) | 4.955 | 10.311 | 7.004 | 2.154 | 1.303 | 14.458 | 2.915 | 4.213 | 5.128 | 4.779 | 5.864 | 8.039 | 9.845 | 5.348 |
| *Podocarpidites ellipticus* Cookson 1947 | Podocarpaceae (*Podocarpus*) | 0 | 7.038 | 9.728 | 0.308 | 0.651 | 7.229 | 0.777 | 5.899 | 5.128 | 2.941 | 5.705 | 3.537 | 15.026 | 5.348 |
| *Podocarpidites rugulosus* Romero 1977 | Podocarpaceae (*Podocarpus*) | 0 | 0.164 | 0 | 0 | 0 | 0 | 0 | 0.281 | 0.321 | 0 | 0.158 | 0 | 5.181 | 0 |
| *Podocarpidites* spp. | Podocarpaceae (*Podocarpus*) | 4.955 | 1.309 | 3.113 | 0.308 | 0.326 | 1.205 | 12.051 | 0.281 | 1.603 | 2.574 | 2.219 | 2.894 | 7.772 | 13.369 |
| *Trisaccites microsaccatum* Couper 1953 | Extinct (Podocarpaceae) | 0 | 1.473 | 2.335 | 0 | 0 | 0 | 0 | 0.843 | 0.321 | 1.287 | 0.158 | 1.286 | 0 | 0 |
| *Vitreisporites pallidus* (Reissinger) Nilsson 1958 | Extinct (Pteridospermatophyta) | 0 | 0.491 | 1.556 | 0 | 0 | 0 | 0 | 0 | 0 | 0 | 0 | 0 | 0 | 0 |
| Angiosperm pollen |  |  |  |  |  |  |  |  |  |  |  |  |  |  |  |
| *Arecipites minutiscabratus* McIntyre 1968 | Arecaceae | 18.018 | 2.128 | 2.724 | 0.308 | 0.651 | 4.096 | 1.458 | 0.843 | 2.564 | 11.581 | 5.547 | 7.074 | 8.290 | 1.872 |
| *Arecipites* sp. | Arecaceae | 0 | 0.164 | 0 | 0 | 0 | 0.964 | 0.194 | 0.281 | 0.321 | 0 | 0 | 0 | 0 | 0 |
| *Bombacacidites* cf. *isoreticulatus* McIntyre 1968 | Malvaceae (Bombacoideae) | 0 | 0 | 0 | 0 | 0 | 0.241 | 0 | 0.281 | 0.321 | 0 | 0.317 | 0 | 0 | 0 |
| *Bombacacidites* sp. 1 | Malvaceae (Bombacoideae) | 0 | 0 | 0.389 | 0 | 0.326 | 0 | 0 | 0 | 0.321 | 0 | 0 | 0 | 0 | 0 |
| cf. *Beaupreaidites* sp. | Proteaceae (*Beauprea*) | 0 | 0 | 0.389 | 0 | 0 | 0 | 0 | 0 | 0 | 0 | 0.158 | 0 | 0 | 0 |
| cf. *Cricotriporites* | Celtidaceae? | 0 | 0 | 0 | 0.308 | 0 | 1.205 | 0.389 | 0 | 0.321 | 0 | 0.158 | 0 | 0 | 0.267 |
| cf. *Forcipites?* *longus* (Stover & Evans) Dettmann & Jarzen 1988 | Unknown angiosperm | 0 | 0 | 0.389 | 0.308 | 0 | 0 | 0 | 0 | 0 | 0 | 0 | 0 | 0 | 0 |
| *Clavatipollenites* sp. | Chloranthaceae | 1.351 | 0.164 | 0 | 0 | 0 | 0.241 | 0.097 | 0.281 | 0.321 | 0.551 | 0.475 | 0.322 | 1.036 | 0.267 |
| *Cranwellipollis* sp. cf. *C.* *apiculatus* Stover & Partridge 1982 | Proteaceae | 0 | 0 | 0 | 0 | 0.326 | 0 | 0 | 0 | 0 | 0 | 0 | 0 | 0 | 0 |
| *Echitriporites* sp. | Unknown angiosperm | 0 | 0 | 0 | 1.538 | 0.326 | 0 | 0.680 | 0.281 | 0.321 | 0 | 0 | 0.322 | 0 | 0 |
| *Ericipites* *microtectatum* Archangelsky & Zamaloa 1986 | Ericaceae/Epacridaceae | 0 | 0 | 0.389 | 0 | 0 | 0 | 0 | 0 | 0 | 0.368 | 0.475 | 0.322 | 0 | 0.267 |
| *Gemmatricolpites* cf. *subsphaericus* Archangelsky 1973 | Aquifoliaceae | 0 | 0 | 0 | 0 | 0 | 0.241 | 0 | 0 | 0 | 0.735 | 0 | 0 | 0 | 0 |
| *Gemmatricolpites* sp. 1 | Aquifoliaceae? | 0 | 0 | 0 | 0.308 | 0.326 | 0 | 0 | 0.281 | 0.321 | 0 | 0.158 | 0 | 0 | 0.267 |
| *Haloragacidites harrisii* (Couper) Harris 1971 | Casuarinaceae | 0 | 0 | 0.389 | 1.538 | 1.954 | 1.205 | 0.972 | 0.562 | 0.641 | 0.919 | 0.317 | 0.643 | 0 | 0.267 |
| *Ilexpollenites clifdenensis* McIntyre 1968 | Aquifoliaceae | 1.351 | 0 | 0 | 0 | 0 | 0.241 | 0.097 | 0.281 | 0.321 | 0 | 0.317 | 0 | 1.554 | 0.802 |
| *Lewalanipollis senectus* Dettmann & Jarzen 1996 | Proteaceae | 0.901 | 0.982 | 0.389 | 0.923 | 1.629 | 1.687 | 0.486 | 0 | 0.321 | 1.103 | 0.158 | 1.929 | 0 | 0 |
| *Lewalanipollis* sp. | Proteaceae | 0 | 0 | 0 | 0 | 0 | 0 | 0.097 | 0 | 0 | 0 | 0 | 0 | 0 | 0 |
| *Liliacidites* cf. *crassibaculatus* Freile 1972 | Liliaceae | 0 | 0.164 | 0.389 | 0 | 0 | 0 | 0 | 0 | 0 | 0 | 0 | 0 | 0 | 0 |
| *Liliacidites regularis* Archangelsky 1973 | Liliaceae | 3.153 | 2.128 | 0.778 | 0 | 0 | 0 | 0 | 0 | 0 | 0 | 0 | 0 | 0 | 0 |
| *Liliacidites variegatus*  Couper 1953 | Liliaceae | 4.054 | 1.964 | 1.167 | 0 | 0 | 0 | 0 | 0.281 | 0 | 0.368 | 0.158 | 0.322 | 0 | 0 |
| *Liliacidites vermireticulatus* Archangelsky & Zamaloa 1986 | Liliaceae | 0 | 0.327 | 0 | 0 | 0 | 0.241 | 0.097 | 0 | 0 | 0.184 | 0.317 | 0.322 | 0 | 0 |
| *Longapertites* aff. v*aneendenburgi* Geermerad Hooping & Muller 1968 | Arecaceae | 1.351 | 0.982 | 2.335 | 0 | 0 | 0.482 | 0.583 | 1.124 | 0 | 0.184 | 0.634 | 0.322 | 0 | 0 |
| *Margocolporites* sp. | Fabaceae? | 0 | 0 | 0 | 0 | 0 | 0.241 | 0 | 0.281 | 0.321 | 0.368 | 0 | 0 | 0 | 0 |
| *Nothofagidites dorotensis* Romero 1973 | *Nothofagus* (*Brassospora*) | 0 | 0 | 0 | 0.308 | 0 | 0 | 0 | 2.528 | 3.526 | 1.287 | 0.792 | 1.929 | 3.627 | 1.337 |
| *Nothofagidites fuegiensis* Menéndez & Caccavari 1975 | *Nothofagus* (*Fuscospora*) | 0 | 0 | 0 | 0 | 0 | 0 | 0 | 0.281 | 3.205 | 3.493 | 1.743 | 5.788 | 1.554 | 1.337 |
| *Nothofagidites saraensis* Menéndez & Caccavari 1975 | *Nothofagus* (*Nothofagus*) | 0 | 0.327 | 0 | 0 | 0 | 0 | 0 | 0.843 | 0 | 0 | 0.158 | 0 | 0 | 0.267 |
| *Nothofagidites* sp. 1 | *Nothofagus* (extinct) | 0 | 0 | 0 | 0 | 0 | 0 | 0.097 | 0.281 | 0.321 | 0.368 | 0 | 0.322 | 0 | 0 |
| *Peninsulapollis* *askiniae* Dettmann & Jarzen 1988 | Unknown angiosperm | 0 | 0 | 1.556 | 0 | 0 | 0.241 | 0 | 0 | 0 | 0 | 0 | 0 | 0 | 0 |
| *Peninsulapollis* cf. *truswelliae* Dettmann & Jarzen 1988 | Proteaceae? (*Beauprea*?) | 1.802 | 0.818 | 0.389 | 0.923 | 0.326 | 0 | 0 | 0.281 | 0.321 | 0 | 0.158 | 0.643 | 0 | 0.267 |
| *Peninsulapollis gillii* (Cookson) Dettmann & Jarzen 1988 | Proteaceae (*Beauprea*) | 4.054 | 1.964 | 1.946 | 4 | 4.56 | 4.096 | 5.053 | 2.247 | 0.962 | 3.125 | 2.060 | 4.180 | 2.073 | 0.535 |
| *Peninsulapollis* sp. 2 | Proteaceae | 0 | 0.327 | 0 | 0 | 0 | 0 | 0.194 | 0 | 0 | 0 | 0.158 | 0 | 0.518 | 0 |
| *Periporopollenites pallidus* Truswell & Owens 1988 | Trimeniaceae? | 0 | 0 | 0 | 0 | 0 | 0 | 0 | 0 | 0 | 0.184 | 0 | 0 | 0 | 0 |
| *Propylipollis ambiguus* (Stover) Dettmann & Jarzen 1996 | Proteaceae (*Telopea*) | 0.450 | 0.164 | 0.778 | 0 | 0 | 0 | 0 | 0 | 0 | 0 | 0 | 0 | 0 | 0 |
| *Propylipollis* cf. *tenuiexinus* (Stover) Dettmann & Jarzen. 1996 | Proteaceae | 0 | 0 | 0 | 0 | 0 | 0 | 0 | 0.281 | 0.321 | 0 | 1.585 | 0 | 0.518 | 0 |
| *Propylipollis reticuloscabratus* (Harris) Martin & Harris 1974 | Proteaceae | 0 | 0 | 0 | 0 | 0 | 0 | 0 | 0 | 0 | 0 | 0.158 | 0 | 0 | 0 |
| *Propylipollis* sp. | Proteaceae | 0 | 1.309 | 3.113 | 0 | 0 | 0 | 0 | 0 | 0 | 0 | 0 | 0 | 0 | 0 |
| *Proteacidites angulatus* Stover 1973 | Proteaceae | 0.450 | 0 | 0.389 | 0 | 0.651 | 0 | 0.194 | 0.281 | 0.321 | 0.919 | 0 | 0 | 0.518 | 0 |
| *Proteacidites* sp. A | Proteaceae | 1.351 | 0 | 0.389 | 0 | 0 | 0 | 0 | 0 | 0 | 0.735 | 0.158 | 0 | 0 | 1.070 |
| *Proteacidites* sp. B | Proteaceae | 1.351 | 0.982 | 0 | 0 | 0 | 0 | 0 | 0 | 0 | 0 | 0 | 0 | 0 | 0 |
| *Proteacidites* sp. C | Proteaceae | 0 | 0 | 0 | 1.538 | 0.326 | 1.446 | 1.944 | 0.562 | 0.641 | 0.184 | 0.317 | 0.643 | 0 | 0.535 |
| Proteacidites sp. D | Proteaceae | 0 | 0 | 0 | 0 | 0 | 0 | 0 | 0 | 0 | 0 | 0 | 0 | 0 | 0.267 |
| *Psilatricolpites* *inargutus* (McIntyre) Archangelsky 1973 | Unknown angiosperm | 0 | 0 | 0.389 | 1.231 | 7.166 | 0.723 | 1.458 | 0.281 | 0.962 | 0 | 0.158 | 0 | 0 | 0 |
| *Psilatricolporites salamanquensis* Archangelsky & Zamaloa 1986 | Unknown angiosperm | 0 | 0 | 0 | 0 | 0 | 0 | 0 | 1.124 | 0.641 | 0 | 0 | 0 | 0 | 0 |
| *Psilatricolporites* sp. 1 | Unknown angiosperm | 6.306 | 3.110 | 2.335 | 2.462 | 0.977 | 0.241 | 0.389 | 0.843 | 0 | 0.735 | 0.792 | 1.608 | 0 | 0.535 |
| *Psilatricolporites* sp. 2 | Unknown angiosperm | 0 | 0 | 0.389 | 0 | 0 | 0 | 0 | 0 | 0 | 0 | 0 | 0 | 0 | 0.535 |
| *Quadraplanus brossus* Stover 1973 | Unknown angiosperm | 0 | 0 | 0.389 | 0 | 0 | 0 | 0 | 0 | 0 | 0 | 0 | 0 | 0 | 0 |
| *Retitricolporite chubutensis*Archangelsky 1973 | Unknown angiosperm | 0 | 0 | 0.389 | 0 | 0.326 | 0.482 | 0.583 | 0.281 | 0 | 0.368 | 0 | 0 | 0 | 0 |
| *Retitricolporites* cf. *R.* *minutiformis* Truswell & Owen 1988 | Unknown angiosperm | 0 | 0 | 0 | 0 | 3.583 | 0 | 0.486 | 0.281 | 0 | 1.654 | 0.158 | 0 | 0 | 0 |
| *Retitricolporites* sp. 1 | Unknown angiosperm | 0.901 | 0.491 | 1.167 | 6.154 | 7.818 | 2.892 | 3.207 | 1.685 | 5.128 | 7.904 | 3.328 | 8.039 | 2.073 | 3.743 |
| *Rhoipites minusculus*Archangelsky 1973 | Unknown angiosperm | 0 | 0 | 0 | 0 | 0.326 | 0 | 0 | 1.124 | 1.923 | 0 | 1.743 | 1.929 | 0 | 0 |
| *Rosannia manika* Srivastava emend. Srivastava & Braman 2010 | Lactoridaceae (*Lactoris*) | 0 | 0 | 0.389 | 0 | 0 | 0 | 0.194 | 0 | 0 | 0 | 0 | 0 | 0 | 0 |
| *Rousea microreticulata* Archangelsky & Zamaloa 1986 | Unknown angiosperm | 0 | 0 | 0 | 0 | 0.326 | 0 | 0 | 0.281 | 0 | 1.471 | 0 | 0 | 0 | 0 |
| *Rousea patagonica* Archangelsky 1973 | Unknown angiosperm | 0 | 0 | 0 | 0 | 0 | 0.482 | 0.097 | 0.562 | 0.321 | 1.654 | 0.317 | 0.643 | 0 | 0.267 |
| *Senipites* *tercrassata* Archangelsky 1973 | Symplocaceae | 0 | 0 | 0.778 | 0.923 | 1.303 | 0.482 | 1.069 | 0.281 | 0.321 | 0.184 | 0.158 | 0 | 0 | 0 |
| *Sparganiaceaepollenites* sp. | Sparganiaceae/Thyphaceae | 0.450 | 0.491 | 0.389 | 0 | 0 | 0.482 | 0.097 | 0.281 | 0.321 | 0.184 | 0.158 | 0 | 0 | 0 |
| *Spinitricolpites* sp. | Unknown angiosperm | 0 | 0 | 0.389 | 0 | 0 | 0 | 0 | 0.281 | 0 | 0 | 0 | 0 | 0 | 0 |
| *Spinizonocolpites hialinus*Archangelsky & Zamaloa 1986 | Arecaceae (*Nypa*) | 0 | 0.327 | 0.389 | 0 | 0.326 | 0.241 | 0 | 0 | 0.321 | 0.551 | 0.317 | 0.322 | 0 | 0.267 |
| *Striatricolporites gamerroi* Archangelsky 1973 | Anacardiaceae | 0 | 0 | 0 | 0 | 0 | 0 | 0 | 0.281 | 0.321 | 0.368 | 0 | 0 | 0 | 0 |
| *Triatriopollenites lateflexus* Archangelsky 1973 | Proteaceae? | 0.450 | 1.146 | 0.389 | 0.308 | 2.280 | 0.482 | 0.389 | 0.281 | 0.962 | 2.757 | 2.060 | 1.608 | 0 | 0.535 |
| *Triatriopollenites* sp. 1 | Proteaceae? | 0 | 0 | 0.389 | 0.923 | 0 | 0 | 0 | 0 | 0 | 0.184 | 0 | 0 | 0 | 0 |
| *Triatripollenites bertelsii* Archangelsky 1973 | Proteaceae? | 0.450 | 0 | 0 | 0 | 1.303 | 0.241 | 0 | 0.281 | 0.321 | 1.838 | 1.268 | 0.965 | 0.518 | 0.267 |
| *Tricolpites reticulatus* Couper 1953 | Gunneraceae (*Gunnera*) | 0.901 | 0.491 | 0.389 | 0.308 | 0.651 | 0.241 | 0.194 | 0 | 0.321 | 0.368 | 0.158 | 0.322 | 0 | 0.267 |
| *Tricolpites* sp. 1 | Unknown angiosperm | 0 | 0 | 0 | 0 | 0 | 0 | 0 | 0.281 | 0.962 | 2.206 | 0.317 | 0.322 | 0 | 0 |
| *Tricolpites* sp. 2 | Unknown angiosperm | 0 | 0 | 0 | 0 | 0 | 0 | 0 | 0 | 0 | 0 | 0.158 | 0.322 | 1.036 | 0.802 |
| *Tricolporites* sp. 3 | Unknown angiosperm | 0 | 0 | 0 | 0 | 0 | 0 | 0 | 0 | 0.321 | 0.919 | 0.634 | 0.322 | 0 | 0 |
| *Triorites* cf. *T.* *orbiculatus* McIntyre 1968 | Unknown angiosperm | 0 | 0 | 0 | 0 | 0 | 0 | 0 | 0.281 | 0 | 0.184 | 0.158 | 0.322 | 0 | 0 |
| *Triporopollenites minor* (Couper) Barreda 1997 | Proteaceae | 1.351 | 0.818 | 0.778 | 2.462 | 0.326 | 0.723 | 0.292 | 0 | 0 | 3.676 | 2.060 | 0 | 2.073 | 0.802 |
| *Tubulifloridites lilliei* (Couper) Farabee & Canright 1986 | Unknown angiosperm | 0 | 0 | 0.778 | 0 | 0 | 0 | 0 | 0 | 0 | 0 | 0 | 0 | 0 | 0 |
| *Ulmoideipites patagonicus* Archangelsky 1973 | Ulmaceae | 0 | 0 | 0 | 0 | 0 | 0 | 0 | 0 | 0.321 | 0 | 0 | 0 | 0 | 0 |
