# Supplementary material for: Cretaceous/Paleogene Floral Turnover in Patagonia: Drop in Diversity, Low Extinction, and a Classopollis Spike
Source: PLoS One. 2012 Dec 17;7(12):e52455. doi: 10.1371/journal.pone.0052455 (PMC3524134; doi:10.1371/journal.pone.0052455)
Supplement: Table S2 — (DOCX) [file pone.0052455.s002.docx]

| Fossil taxon | Proportional abundances | | | | | | | | | | | | | |
| --- | --- | --- | --- | --- | --- | --- | --- | --- | --- | --- | --- | --- | --- | --- |
|  | M1 | M2 | M3 | D1 | D2 | D3 | D4 | D5 | D6 | D7 | D8 | D9 | D10 | D11 |
| Bryophyte spores | 1.37 | 0.49 | 1.18 | 0.31 | 0.33 | 0.48 | 0.10 | 1.71 | 1.96 | 0.37 | 0.80 | 0 | 0 | 1.36 |
| Lycophyte spores | 0.46 | 0.82 | 1.97 | 0.62 | 0.98 | 0 | 0.49 | 0.86 | 0.65 | 0.37 | 0.48 | 0.32 | 0 | 0.27 |
| Pteridophyte spores | 35.62 | 48.03 | 38.58 | 32 | 30.39 | 25.3 | 32.85 | 31.14 | 19.93 | 22.61 | 37.54 | 28.3 | 20.73 | 27.1 |
| Gymnosperms | 12.79 | 29.28 | 30.71 | 40.62 | 30.39 | 49.64 | 45.48 | 46.57 | 49.35 | 22.79 | 31.31 | 29.58 | 53.37 | 54.74 |
| Angiosperms | 51.14 | 21.88 | 28.74 | 26.77 | 38.24 | 24.58 | 21.09 | 21.43 | 30.07 | 53.86 | 30.67 | 41.8 | 25.91 | 17.89 |

**Table S2**. Proportional abundance of the main floristic groups
